# Effects of the Mediterranean Diet on Cardiovascular Outcomes—A Systematic Review and Meta-Analysis

**DOI:** 10.1371/journal.pone.0159252

**Published:** 2016-08-10

**Authors:** Thaminda Liyanage, Toshiharu Ninomiya, Amanda Wang, Bruce Neal, Min Jun, Muh Geot Wong, Meg Jardine, Graham S. Hillis, Vlado Perkovic

**Affiliations:** 1 The George Institute for Global Health, Sydney Medical School, University of Sydney, PO Box M201, Missenden Road, Sydney, NSW, 2050, Australia; 2 Armadale Kelmscott Memorial Hospital, South Metropolitan Health Service, Armadale, WA, 6112, Australia; 3 Department of Medicine, Division of Nephrology, University of Calgary, Calgary, Alberta, Canada; 4 Concord Repatriation General Hospital, Hospital Road, Concord, Sydney, NSW, 2139, Australia; University of British Columbia, CANADA

## Abstract

**Background:**

A Mediterranean dietary pattern is widely recommended for the prevention of chronic disease. We sought to define the most likely effects of the Mediterranean diet on vascular disease and mortality.

**Methods:**

We searched MEDLINE, EMBASE and the Cochrane Central Register without language restriction for randomized controlled trials comparing Mediterranean to control diets. Data on study design, patient characteristics, interventions, follow-up duration, outcomes and adverse events were sought. Individual study relative risks (RR) were pooled to create summary estimates.

**Results:**

Six studies with a total of 10950 participants were included. Effects on major vascular events (n = 477), death (n = 693) and vascular deaths (n = 315) were reported for 3, 5 and 4 studies respectively. For one large study (n = 1000) there were serious concerns about the integrity of the data. When data for all studies were combined there was evidence of protection against major vascular events (RR 0.63, 95% confidence interval 0.53–0.75), coronary events (0.65, 0.50–0.85), stroke (0.65, 0.48–0.88) and heart failure (0.30, 0.17–0.56) but not for all-cause mortality (1.00, 0.86–1.15) or cardiovascular mortality (0.90, 0.72–1.11). After the study of concern was excluded the benefit for vascular events (0.69, 0.55–0.86) and stroke (0.66, 0.48–0.92) persisted but apparently positive findings for coronary events (0.73, 0.51–1.05) and heart failure (0.25, 0.05–1.17) disappeared.

**Conclusion:**

The Mediterranean diet may protect against vascular disease. However, both the quantity and quality of the available evidence is limited and highly variable. Results must be interpreted with caution.

## Introduction

Cardiovascular disease (CVD) is a leading cause of mortality worldwide accounting for one quarter of the estimated 52 million deaths in 2010[1,2]. The burden of CVD varies between regions and a proportion of this variability may be due to different dietary patterns[1,3].

Populations in the Mediterranean region have been reported to experience lower morbidity and mortality rates from CVD compared with northern European populations[4], which has been attributed to their consumption of a Mediterranean diet high in fruits and vegetables, nuts and cereals, fish, olive oil, with moderate red wine consumption and minimal amounts of red meat and dairy products[5–7]. In addition, the Mediterranean diet has been associated with beneficial effects on CVD risk factors such as glycaemia, blood pressure and lipid levels[8–10]. Two recent systematic reviews have showed some beneficial effects of the Mediterranean diet on the metabolic syndrome and its constituents but neither have explored the effects on vascular events or mortality[11,12].

Although there are randomized controlled trials (RCTs) assessing the effect of the Mediterranean diet on the primary or secondary prevention of CVD[13,14] the largest of these trials recorded just a few hundred events. There is also concern that one of the key trials supporting the beneficial effects of the Mediterranean diet was seriously methodologically compromised[15]. There is, therefore, real uncertainty about the data underpinning the value of the Mediterranean diet to human health. We undertook this systematic review and meta-analysis to better define the effects of the Mediterranean diet on cardiovascular events and mortality.

## Materials and Methods

### Data sources and searches

We performed a systematic review of the literature according to the PRISMA statement for the conduct of meta-analyses of intervention studies (www.prisma-statement.org) (S1 File). Relevant studies were identified by searching the following data sources: MEDLINE via Ovid (from inception through February 2014), EMBASE (from inception through February 2014) and the Cochrane Library database (Cochrane Central Register of Controlled Trials; no date restriction), using relevant text words and medical subject headings (S2 File). The search was limited to randomised controlled trials (RCTs) without language restriction. Reference lists from identified trials and review articles were manually scanned to identify any other relevant studies. The clinicaltrials.gov website was also searched for RCTs that were registered as completed but not yet published.

### Study selection

The literature search, data extraction and quality assessment were conducted independently by two authors using a standardised approach (TL and AW). All completed RCTs assessing the effects of a Mediterranean diet compared to any control diet involving adults, with follow up period longer than three months, that reported one or more of the relevant outcomes were eligible for inclusion. Although there is considerable variability, a diet with non-restricted fat intake and with at least two of the following seven components was defined as a Mediterranean style diet for the purposes of this review; 1) use of olive oil as the main cooking oil, 2) moderate red wine consumption, 3) high consumption of nuts and/or legumes, 4) high consumption of grains and cereals, 5) high consumption of fruits and vegetables, 6) high consumption of fish with low consumption of meat and meat products and 7) low to moderate consumption of milk and dairy products[16].

### Data extraction and quality assessment

Published reports were obtained for each trial and standard information about each trial was extracted into a spreadsheet. The data sought included summary metrics for each trial about baseline patient characteristics (age, gender, history of diabetes, history of hypertension, mean systolic and diastolic blood pressure levels, lipid levels, smoking status, body mass index and history of cardiovascular disease), details of the dietary intervention, follow-up duration, outcome events and adverse events. Study quality was judged according to the Cochrane risk of bias assessment tool[17]. Any disagreement in extracted data was adjudicated by a third reviewer (VP).

### Outcomes

We sought data on total cardiovascular events (stroke, myocardial infarction, cardiovascular mortality or a broadly comparable definition reported by the study), coronary events (fatal or non-fatal myocardial infarction, coronary revascularization), cerebrovascular events (fatal or non-fatal stroke), heart failure, total mortality, incidence of type 2 diabetes mellitus, end-stage kidney disease, adverse effects (all adverse effects and serious adverse effects) and quality of life.

### Data synthesis and analysis

Individual study relative risks (RRs) and 95% confidence intervals (CIs) were calculated from event numbers extracted from each trial. In calculating RRs, the total number of patients randomized in each group was used as the denominator. If no events were reported in either treatment or control group of any trial, 0.5 was used as the numerator. For the three arm PREDIMED study[14], the olive oil supplement arm and the nuts supplement arm were jointly considered as the treatment group whereas in the four arm study by Burr et al[18] only the “fish and fruit” arm fulfilled the defined criteria for the Mediterranean diet and this was compared to the “sensible eating” arm. Primary summary estimates of RRs were obtained using a random fixed effects model. Given the small number of included trials, publication bias and percentage of variability across studies attributable to heterogeneity beyond chance were not assessed as these may be unreliable. A two-sided p-value less than 0.05 was considered statistically significant. All analyses were performed with STATA, version 9.2 (Stata, College Station, Texas).

## Results

### Search results and characteristics of included studies

**Fig 1:**
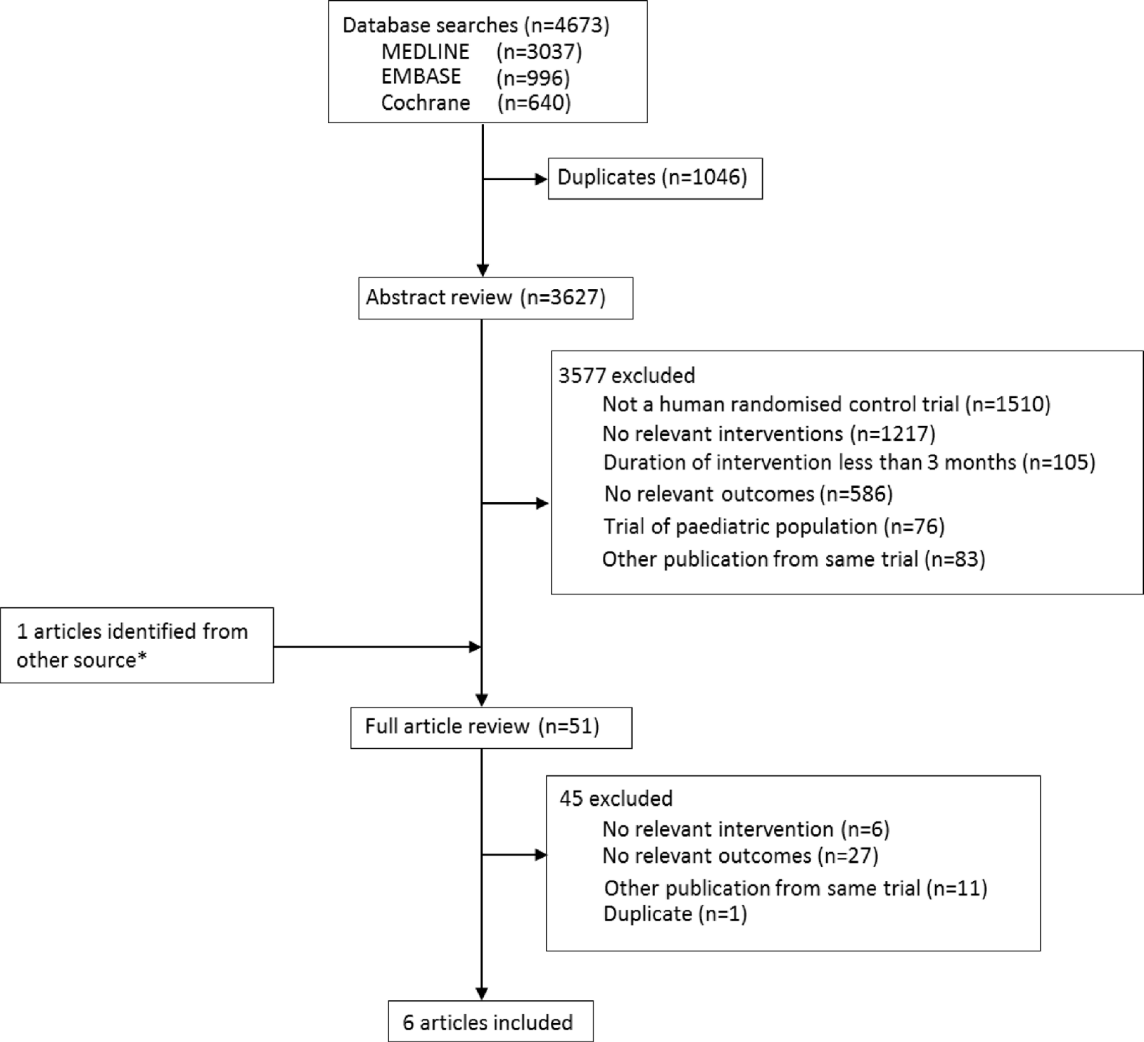
Identification process for eligible studies.

The literature search yielded 4637 articles, of which 51 were reviewed in full text (Fig 1). Of these, six RCTs, involving a total of 10950 participants, met the inclusion criteria. Among these, 477 major cardiovascular events were reported from three trials[13,14,19] and 693 deaths from five trials[13,14,18–20]. The remaining study assessed the effect on quality of life[21]. The majority of the excluded trials did not study the Mediterranean diet as defined in the protocol, studied only individual components of the Mediterranean diet, did not report relevant outcomes, were not original investigations or were duplicate reports (S3 File).

The trials had a sample size that ranged from 48 to 7447 participants (Table 1). Two studies were single-centre[13,20] and four were multi-centre[14,18,19,21]. All studies provided dietary advice to participants and two studies provided food supplements in addition to dietary advice[13,14]. The studies were conducted between March 1988 and December 2010. The mean age of the study participants ranged between 41 and 67 years. One study only enrolled men[18], one study only enrolled women[21] and four studies enrolled both men and women[13,14,19,20]. Primary prevention was the objective of three studies[14,20,21], secondary prevention was the goal of two studies[13,18] and the remaining study included patients with and without a prior history of cardiovascular disease[19].

**Table 1:** Characteristics of the studies included in the systematic review and meta-analysis.

**Author**	**Inclusion criteria**	Treatment Group	Control Group	Setting	Duration of follow up (mean)	Total number of patients	Mean age (yrs)	Male, n (%)	DM n (%)	Primary or secondary prevention	Number of composite CV events #	Number of coronary events	Number of cerebro-vascular events	Number of all-cause deaths
de Lorgeril et al(1994)	Age <70; first MI within 6 mths	Advice on high bread, vegetables, fish and less meat. Margarine supplements as it was considered patients would not accept olive oil as only fat	Prudent western diet advice by attending physician	Single centre France	27 months	605	53.5	549 (90.7)	NR	Secondary	44	22	3	28
Singh et al (2002)	>25yrs with cardiac RF or angina, previous MI	Indo-Mediterranean diet advice (high fruit, vegetables, nuts, whole grains, mustard seed or soy bean oil)	Dietary advice according to NCEP guidelines	Multi centre India	2 years	1000	48.5	897 (89.7)	210 (21)	Primary and secondary	145	93	25	62
Toobert et al (2003)	Post-menopausal women with type 2 DM	Mediterranean diet advice, physical activity, stress management, social support, smoking advice	Usual post-menopausal care	Multi centre USA	6 months	279	NR	0 (0)	NR	Primary	NR	NR	NR	NR
Burr et al (2003)	Males <70yrs with angina	Oily fish, fruit, vegetables and oats diet advice	Sensible eating	Multi centre United Kingdom	3–9 years (range)	1571	61.15	1571 (100)	210 (13.3)	Secondary	NR	NR	NR	251
Ng et al (2011)	>18 yrs with HIV	Advice on high fruit, vegetables, nuts, white meat, canola or olive oil as main cooking oil.	Dietary advice according to NCEP guidelines	Single centre Hong Kong	1 year	48	41	37 (77)	NR	Primary	NR	NR	NR	4
Estruch et al (2013)	Male (55–80 yrs) and female (60–80 yrs) with type 2 DM or ≥3 major cardiac risk factors	Mediterranean diet +extra virgin olive oil; Mediterranean diet + nuts; both intervention groups received supplements	Low fat control diet	Multi centre Spain	4.8 years (median)	7447	67	3165 (42.5)	3614 (48.5)	Primary	288	106	139	348

#defined as per authors or combined cardiac and cerebrovascular endpoints

MI = myocardial infarction; DM = diabetes mellitus; CV = cardiovascular; RF = risk factors; HIV = human immunodeficiency virus; NECP = national cholesterol education programme; USA = United States of America

In regard to study quality (Table 2), four of the six studies concealed the randomized allocation sequence[13,14,19,20], no studies were double-blinded and blinded assessments of outcomes were made in only 3 studies[13,14,19]. For one study subsequent publications raised serious concerns about the integrity such that the veracity of results was significantly in doubt[15,19].

**Table 2:** Quality assessment of included studies.

**Study/author (year)**	Random sequence generation	Allocation concealment	Blinding of participants and personnel	Blinding of outcome assessment	Completion rate (%)(Treatment/Placebo)	Intention-to-treat described	Incomplete outcome data adequately addressed	Selective outcome reporting
Lyon Diet Heart Study, de Lorgeril et al (1994)	Yes	Yes	No	Yes	90.1/87.4	Yes	Yes	No
Indo-Mediterranean Diet Study, Singh et al (2002)	Yes	Yes	No	Yes	98.2/97.8	Yes	Yes	No
Toobert et al (2003)	Yes	No	No	No	84/93.1	Yes	Yes	No
Burr et al (2003)	Yes	No	No	No	100/100	Yes	Yes	No
Ng et al (2011)	Yes	Yes	No	No	68/82.6	Yes	Yes	No
PREDIMED study, Estruch et al (2013)	Yes	Yes	No	Yes	95.1/88.7	Yes	Yes	No

### Effects of Mediterranean diet on vascular outcomes and death

#### Major cardiovascular events

**Fig 2:**
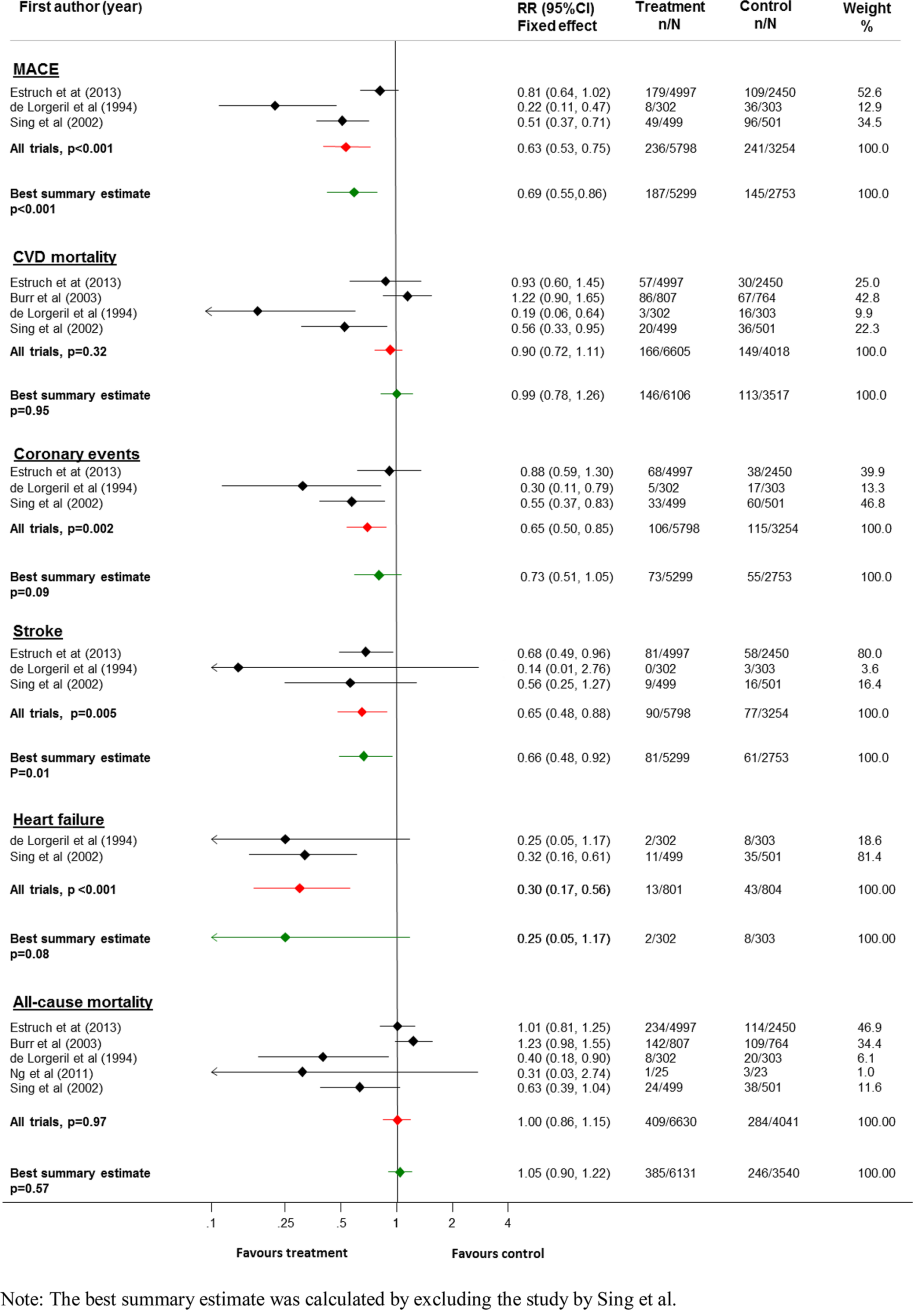
Forrest plot of the included studies showing relative risk of Mediterranean diet compared to control diet by various outcome measures (Fixed effects model analysis).

Effects of the Mediterranean diet were reported for this outcome by three trials[13,14,19] including 9052 participants and 477 events. The Mediterranean diet was associated with a 37% relative reduction in the risk of major cardiovascular events (RR 0**·**63, 95% CI: 0**·**53–0**·**75, p< 0**·**001) compared to control diet. Exclusion of the study with serious concerns about its integrity (n = 1000, 145 events) did not result in a change of overall direction of the effect (RR 0.69, 95% CI 0.55–0.86, p< 0.001) (Fig 2).

#### Fatal outcomes

Data on all-cause mortality were available from 5 trials[13,14,18–20] including 10671 participants and 693 deaths, four of which also separately reported data for cardiovascular mortality (10623 participants and 315 deaths) [13,14,18,19]. The Mediterranean diet had no clear effect on all-cause mortality (RR 1**·**00, 95% CI: 0**·**86–1**·**15, p = 0**·**97) or cardiovascular mortality (RR: 0**·**90, 95% CI: 0**·**72–1**·**11, p = 0**·**32) compared to control (Fig 2). Excluding the trial by Singh et al. further widened the confidence intervals (Fig 2). Across the various subgroups of studies examined (S1 and S2 Figs), greater benefits were suggested in small, single centre studies with incomplete follow-up.

#### Cause-specific cardiovascular outcomes

Three trials[13,14,19] reported 221 coronary events and 167 cerebrovascular events. Use of the Mediterranean diet was associated with a relative risk of 0**·**65 (95% CI 0.50–0**·**85) (Fig 2) for coronary events and 0**·**65 (95% CI 0**·**48–0**·**88) for cerebrovascular events. Only two studies[13,19] involving 1605 participants and reporting 56 events were available to provide an effect estimate for heart failure, showing a relative risk of 0**·**30 (95% CI: 0**·**17–0**·**56). Exclusion of the data from the trial by Singh et al resulted in null findings for all of the cause specific outcomes except cerebrovascular events which continued to remain significant (RR: 0**·**66, 95% CI: 0**·**48–0**·**92, p = 0**·**01) (Fig 2).

#### Effects on other outcomes and adverse events

One study that enrolled 279 participants reported data on quality of life[21]. There was a significant improvement with Mediterranean diet as measured by the Problem Areas in Diabetes (PAID) [22] self-care summary score (p = 0**·**005) but no effect on any other domain of that score or the Medical Outcome Study (MOS -12) short form general health survey[23] (all p>0.05). No data were available on new onset type 2 diabetes mellitus, end-stage kidney disease or adverse events for any of the included studies.

## Discussion

This systematic review and meta-analysis supports the beneficial effects of the Mediterranean diet on cardiovascular outcomes, although the quantity and quality of the available evidence is relatively limited and there is much greater uncertainty about the effects of the Mediterranean diet than is widely understood as a result. There are also no data about adverse outcomes reported by any study, creating difficulties in the understanding of the overall balance between the risks and benefits of the Mediterranean diet. While the completed trials suggest it is likely that the Mediterranean diet protects against certain types of vascular diseases the evidence base falls short of that required to make definitive conclusions or firm recommendations.

Underpinning the rationale for the large-scale outcome trials were the findings of a series of prospective cohort studies[5–7] and recent reviews of those studies that reported moderately decreased risks of all-cause mortality, cardiovascular mortality and the incidence of cardiovascular disease associated with a Mediterranean diet[24,25]. However, observational studies of the effects of diet on cardiovascular disease are highly prone to confounding and it is hard to infer causation from these types of studies. Also in support of protective effects are the data from smaller RCTs examining the effects of Mediterranean diet on intermediate metabolic outcomes. These trials show small to moderate beneficial effects for multiple determinants of vascular risk[26–28]. A recent overview of these RCTs trials identified blood pressure falls of 2.4mmHg (1.2 to 3.5) systolic and 1.6mmHg (1.1 to 2.0) diastolic, an LDL reduction of 2.71 mg/dl (5.03 to 0.39), a reduction in serum triglycerides of 6.14mg/dl (1.93 to 10.35) and an elevation in HDL cholesterol of 1.17mg/dl (0.38 to 1.96)[12].

Despite these encouraging findings, neither the cohort studies nor the RCTs of intermediate outcomes show benefits of a magnitude congruent with the very large point estimates of effect on cardiovascular outcomes proposed by some of the individual trials included in our review. Furthermore, while several other potential mechanisms of benefit such as antioxidant or anti-inflammatory effects have been postulated for the Mediterranean diet[29,30], large trials of intervention strategies specially targeting these pathways have been universally negative[31]. This raises an important question about the plausibility of large beneficial effects of the Mediterranean diet and raises concern about the possible effects of random or systematic errors. In regard to the former, the total volume of data available is small and much less than might typically be available for comparable investigations of the effects of drugs on the same outcomes. In terms of possible systematic errors, the un-masked design of the studies is a substantial weakness inherent in the nature of the intervention and may have led to differential ascertainment and assignment of outcomes across randomised groups. The much greater estimated treatment effects on more subjective outcomes like coronary events, strokes and heart failure compared to the non-significant estimates of effect for the objective outcome of mortality reinforces this possibility.

This review benefits from its comprehensive, systematic approach and the broad range of clinically important endpoints considered (S4 File). It does, however, suffer from a number of limitations. We could not explore heterogeneity across the individual study results for key outcomes in detail and our analyses based upon limited published summary data are restricted in their capacity to investigate this. Heterogeneity seems a possibility not only due to the different nature of the interventions used across the trials but also due to a combination of factors such as differing outcome definitions and varied durations of follow-up. In addition, the fact that larger benefits were seen in smaller, single-centre studies with incomplete follow up raises the possibility of overestimation of benefits by these studies. The absence of any data about the risks of adverse events is also a significant short-coming. Another issue affecting the evidence base supporting the use of the Mediterranean is the concern about the integrity of the trial by Singh et al.[19]. Following a site visit organized by the Lancet to Dr. Singh’s research facility due to concerns raised by multiple parties, including then the editor of BMJ, it was concluded that it was not possible to be sure of the rigor for recording data and its handling. While unable to point to specific evidence to refute the study's results, they raised concerns about the quality of the data likely to be generated from a research facility that lacked access to the kind of highly qualified support staff necessary to conduct a clinical trial to sufficiently high international standards. These concerns have been extensively reported and it is clear that the conduct and reporting of the trial are so weak as to raise serious concerns about the trial results[15]. If the Mediterranean diet was seeking registration or reimbursement under any regulatory assessment scheme it seems very unlikely that the data provided by the Singh trial would be eligible to contribute. Accordingly the best current estimates of the effect of the Mediterranean diet on cardiovascular outcomes are those that exclude the data from that study.

## Conclusion

The American Heart Association/American College of Cardiology[32], the European Society of Cardiology[33] and the National Heart Foundation of Australia (www.heartfoundation.org.au) are three societies that all recommend the Mediterranean diet to reduce cardiovascular risk. All are careful to base this advice on the observed favorable although modest effects of the Mediterranean diet on intermediate cardiovascular risk factors. Clearly, recommendations based upon definitive clinical outcomes would be far preferable, and are supported by the findings of this review, however the strength of conclusions should remain circumspect because of the weakness of the evidence base. It seems unlikely that clinicians and patients fully appreciate the limitations of the data and the extent of the uncertainty. This is an area that warrants another well-conducted, adequately powered trial able to precisely and reliably define the overall balance of benefits and risks associated with the Mediterranean diet.

## Prior Presentations of Results

This work has been presented at the American College of Cardiology Annual Scientific Sessions in 2015 in abstract form. The abstract is available from the following link: http://content.onlinejacc.org/article.aspx?articleid=2198774

## Supporting Information

S1 Fig Subgroup analysis of five studies reporting all-cause mortality. (TIFF)

S2 Fig Subgroup analysis of four studies reporting cardiovascular mortality. (TIFF)

S1 File Study Protocol. (DOCX)

S2 File Search Strategy. (DOCX)

S3 File List of rejected articles after full text review. (RTF)

S4 File PRISMA check list. (DOC)
