# Supplementary figures and images for: Effects of the Mediterranean Diet on Cardiovascular Outcomes—A Systematic Review and Meta-Analysis
Source: PLoS One. 2016 Aug 10;11(8):e0159252. doi: 10.1371/journal.pone.0159252 (PMC4980102; doi:10.1371/journal.pone.0159252)

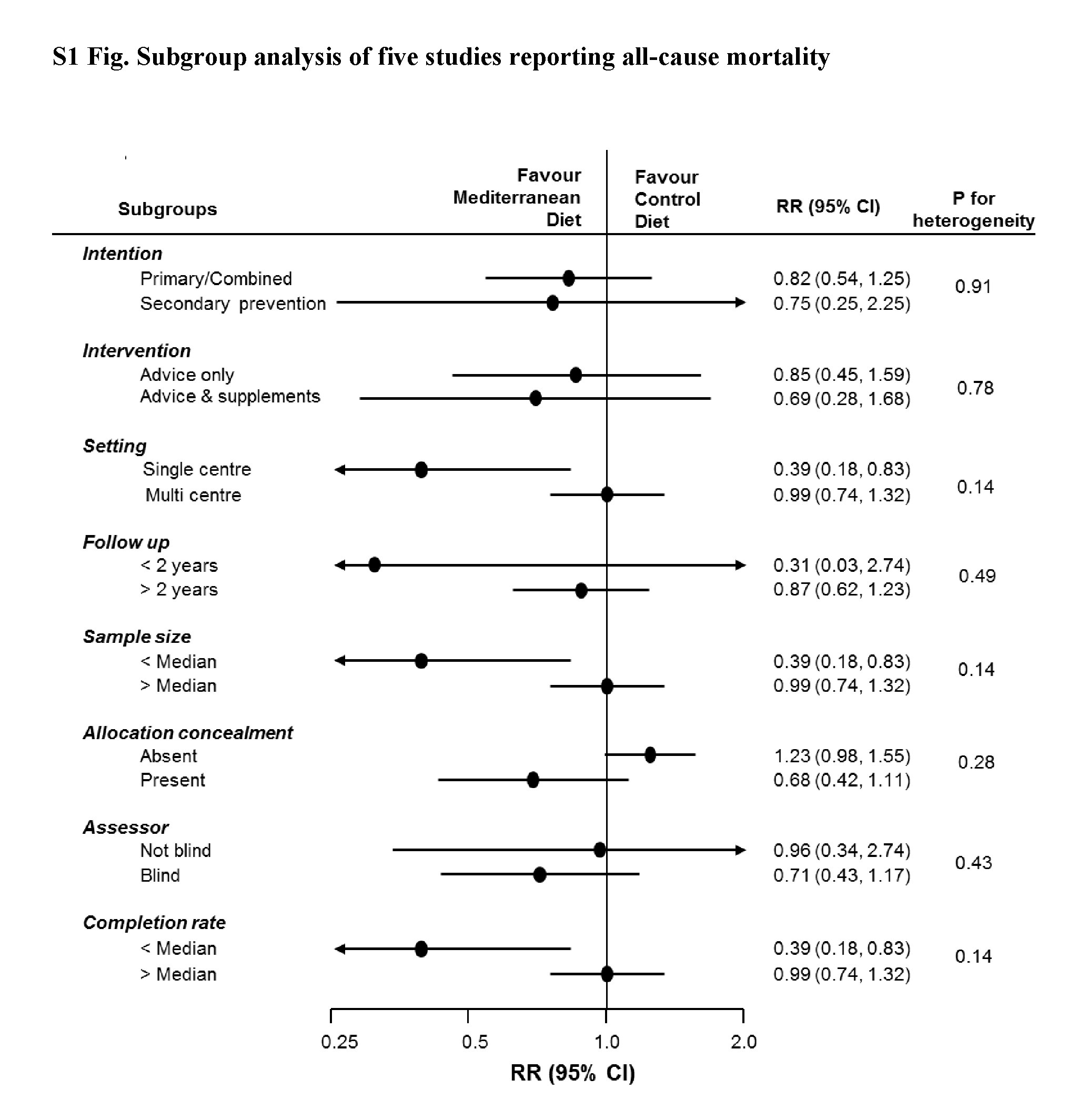

Supplement: S1 Fig — (TIFF) [file pone.0159252.s001.tiff]

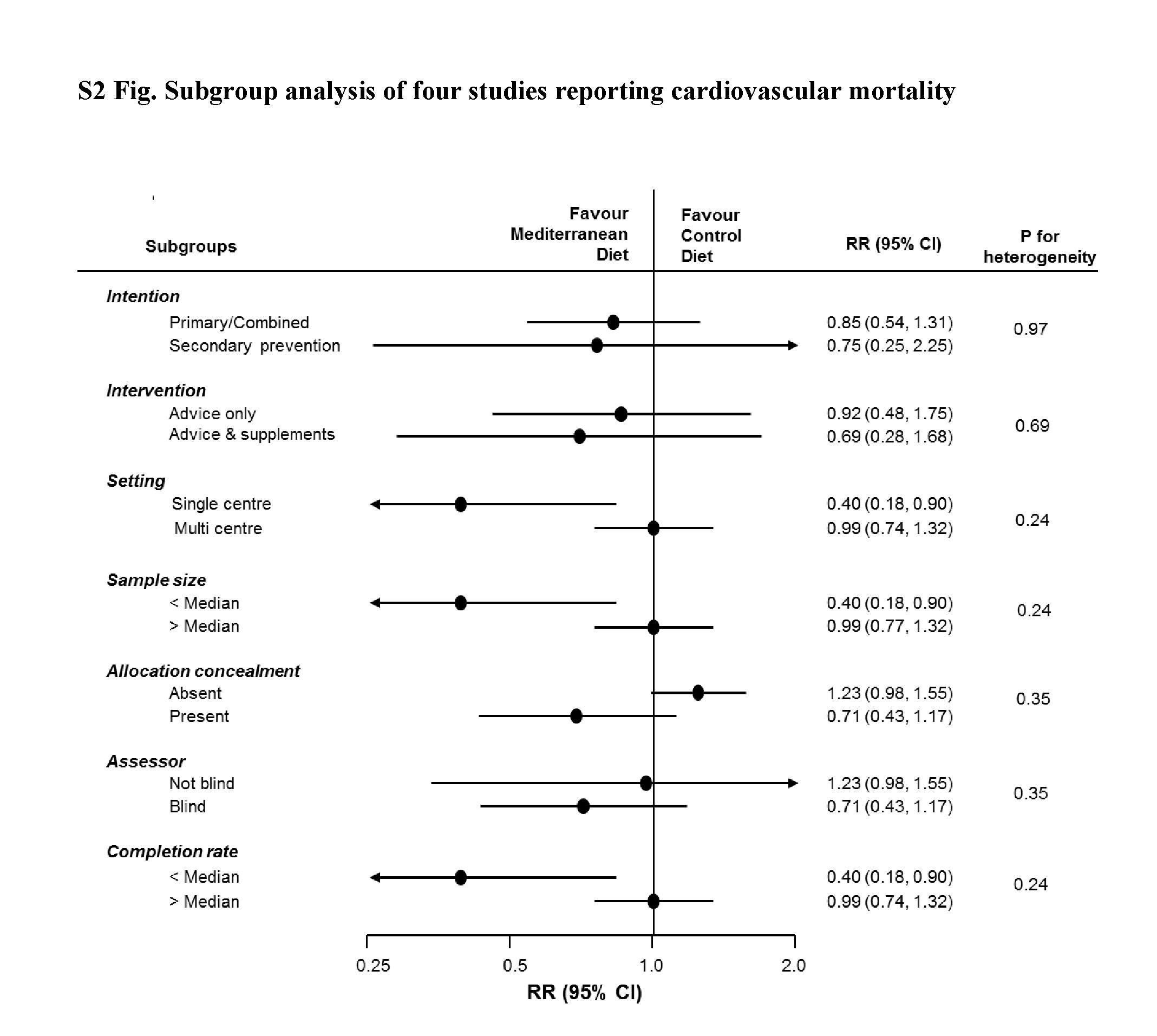

Supplement: S2 Fig — (TIFF) [file pone.0159252.s002.tiff]
