# Supplementary material for: Effects of the Mediterranean Diet on Cardiovascular Outcomes—A Systematic Review and Meta-Analysis
Source: PLoS One. 2016 Aug 10;11(8):e0159252. doi: 10.1371/journal.pone.0159252 (PMC4980102; doi:10.1371/journal.pone.0159252)
Supplement: S1 File — (DOCX) [file pone.0159252.s003.docx]

**S1 File. Effects of Mediterranean diet on cardiovascular outcomes – Protocol for a systematic review and meta-analysis**

Thaminda Liyanage (MMed)^1,2^, Toshiharu Ninomiya (PhD)^1^, Amanda Wang (MBBS)^1^, Bruce Neal (PhD)^1^, Min Jun (PhD)^1,3^, Muh Geot Wong (PhD)^1,2^, Meg Jardine (PhD)^1,4^, Graham S. Hillis (PhD)^1^ and Vlado Perkovic (PhD)^1,2^

Affiliations:

1. The George Institute for Global Health

Sydney Medical School, University of Sydney

PO Box M201, Missenden Road, Sydney

NSW 2050, Australia

1. Royal North Shore Hospital

Reserve Road, St Leonards, Sydney

NSW 2065, Australia

1. Department of Medicine, Division of Nephrology

University of Calgary

Alberta, Canada

1. Concord Repatriation General Hospital

Hospital Road, Concord, Sydney

NSW 2139, Australia

**Background**

Cardiovascular disease (CVD) is one of the leading causes of death worldwide (1). In 2008 it accounted for 30% of total global deaths, including 6.2 million deaths due to stroke and 7.2 million due to coronary heart disease (CHD) (1). The burden of CVD also varies considerably between regions (2, 3). There is a longstanding recognition that diet plays a major role in the etiology of many chronic diseases, thereby contributing to a significant geographical variability in morbidity and mortality rates from chronic disease across different countries and populations worldwide (4).

Populations in the Mediterranean region have been reported to experience lower mortality from CVD compared with northern European populations, possibly as a result of different dietary patterns (5). Several observational studies have suggested reduced mortality and morbidity in regions consuming a Mediterranean diet (6 -13).

Furthermore, the Mediterranean diet has been associated with favorable effects on CVD risk factors. For example, recent studies have reported a decreased incidence of hypertension, diabetes mellitus, and metabolic syndrome with a greater adherence to a Mediterranean dietary pattern (14-17). These findings have been strengthened by recent systematic reviews supporting beneficial effects of the Mediterranean diet on metabolic syndrome and its individual components (18, 19).

There have been a number of randomized controlled trials (RCTs), assessing the efficacy of the Mediterranean diet in the primary or secondary prevention of CVD (20-24). There is considerable variability in the definition of Mediterranean diet and duration of the interventions evaluated and uncertainty regarding the net effect on major outcomes as well as their consistencies. We therefore undertook this systematic review and Meta analysis.

**Objectives**

To conduct a systematic review and a Meta-analysis of RCTs to evaluable the effects of Mediterranean diet in primary and secondary prevention of CV outcomes.

**Methods**

Types of studies

All randomized controlled trials (RCTs) reporting the effects of Mediterranean diet with data available on CV outcomes.

Types of participants

Adult participants with or without CV disease exposed to Mediterranean diet continuously for at least 3 months duration

Types of interventions

Providing specific dietary advice to follow a Mediterranean style diet or provision of dietary supplements relevant to the Mediterranean diet

A diet with non-restricted fat intake and with at least two of the following seven components

will be required to meet our definition of a Mediterranean style diet (25-28).

(1) High monounsaturated/saturated fat ratio (use of olive oil as main cooking ingredient).

(2) Low to moderate red wine consumption.

(3) High consumption of legumes.

(4) High consumption of grains and cereals.

(5) High consumption of fruits and vegetables.

(6) Low consumption of meat and meat products and increased consumption of fish.

(7) Moderate consumption of milk and dairy products.

Types of outcome measures

- CV events – stroke, MI or CV death (or comparable definition by study authors)
- Coronary events – fatal/non fatal MI, coronary revascularization, heart failure
- Cerebrovascular events – fatal/non-fatal stroke
- All-cause mortality
- Incidence of type 2 diabetes
- Adverse effects – all AE, serious AE
- End stage kidney disease (ESKD)
- Quality of life

Search methods for identification of studies

We will search relevant studies from the following sources.

1. Cochrane Central Register of Controlled Trials CENTRAL (Inception-February 2014)
2. MEDLINE OVID SP (Inception-February 2014)
3. EMBASE OVID SP (Inception-February 2014)
4. ClinicalTrials.gov
5. Other resources

- Experts in the field will be contacted for unpublished and ongoing trials
- Authors will be contacted where necessary for additional information
- Reference lists of review articles and relevant studies

Selection of studies

Two authors (TL & AW) will independently review the abstracts of all studies from the initial search. Those that meet the inclusion criteria will be collated. Two authors will independently apply the inclusion criteria to each full text article. Any discrepant assessments will be resolved in discussion with a third author (VP) by consensus.

Data extraction and management

Two authors (TL & AW) will independently extract information using a standardized data collection form. These data will be extrapolated from tables and graphs in published papers. Extracted data will include type of intervention and their duration and outcomes measures.

Assessment of risk of bias in included studies

The following items will be independently assessed for RCTs by two authors using the risk of bias assessment tool (29).

- - Was there adequate sequence generation (selection bias)?
  - Was allocation adequately concealed (selection bias)?
  - Was knowledge of the allocated interventions adequately prevented during the study (detection bias)?

Participants and personnel

Outcome assessors

- - Were incomplete outcome data adequately addressed (attrition bias)?
  - Are reports of the study free of suggestion of selective outcome reporting (reporting bias)?
  - Was the study apparently free of other problems that could put it at a risk of bias?

Measures of treatment effect

We will collect data on CV events (stroke, MI or CV death or comparable definition by study authors), coronary events (fatal/non-fatal MI, coronary revascularization, heart failure), cerebrovascular events (fatal/non-fatal stroke), all-cause mortality, incidence of type 2 diabetes, adverse effects (all AE, serious AE), ESKD and quality of life.

Dealing with missing data

If information is missing from a study, we will contact the authors to obtain the necessary information.

Data synthesis and analysis

Individual study relative risks (RRs) and 95% confidence intervals (CIs) will be calculated from event numbers extracted from each trial before data pooling. In calculating risk ratios, the total number of patients randomized in each group will be used as the denominator. Summary estimates of relative risk ratios will be obtained using a random effects model. The percentage of variability across studies attributable to heterogeneity beyond chance will be estimated using the I^2^ statistic (30). Potential publication bias is assessed using the Egger test and represented graphically using Begg funnel plots of the natural log of the RR versus its standard error (31). Potential heterogeneity in estimates of treatment effect will be explored using univariate meta-regression (30) and by comparing summary results obtained from subsets of studies grouped by number of patients, number of mortality events, publication year, intervention used, diabetic status, baseline morbidity and study quality. A cumulative meta-analysis will be performed to identify any trends in the effect of Mediterranean diet over time. A two-sided p-value less than 0.05 Is considered statistically significant for all analyses. All statistical analyses will be performed with STATA, version 9.2 (Stata, College Station, Texas).

Sensitivity analysis

If a sufficient number of studies (more than 10) are included, we will explore the robustness of the results by performing a sensitivity analysis by incrementally including studies with a high risk of bias.

**References**

1. World Health Organisation. Cardiovascular Diseases (CVDs). Fact Sheet Number 317 September 2011.
2. Müller-Nordhorn J, Binting S, Roll S,Willich S. An update on regional variation in cardiovascular mortality within Europe. *European Heart Journal* 2008;**29**:1316–1326.
3. Reddy KS, Yusuf S. Emerging Epidemic of Cardiovascular Disease in Developing Countries. *Circulation* 1998;**97**: 596–601.
4. WHO Study Group. *Diet, nutrition and the prevention of chronic diseases. WHO Technical Report Series 916*. Geneva: WHO, 2003.
5. Keys A,Menotti A, KarvonenMJ, et al.The diet and 15-year death rate in the Seven Countries Study. *Am J Epidemiol* 1986;**124**:903–915.
6. Benetou V, Trichopoulou A, Orfanos P, et al.Conformity to traditional Mediterranean diet and cancer incidence: the Greek EPIC cohort. *Br J Cancer* 2008;**99**:191–5.
7. Buckland G, Gonzalez CA, Agudo A, et al.Adherence to the Mediterranean diet and risk of coronary heart disease in the Spanish EPIC Cohort Study. *Am J Epidemiol* 2009;**170**:1518–29.
8. Feart C, Samieri C, Rondeau V, et al.Adherence to a Mediterranean diet, cognitive decline, and risk of dementia. *JAMA* 2009;**302**:638–48.
9. Fung TT, Rexrode KM, Mantzoros CS, Manson JE, Willett WC, Hu FB. Mediterranean diet and incidence of and mortality from coronary heart disease and stroke in women. *Circulation* 2009;**119**:1093–1100.
10. Knoops KTB, de Groot LCPGM, Kromhout D, et al.Mediterranean diet, lifestyle factors, and 10-year mortality in elderly European men and women. *JAMA* 2004;**292**:1433–9.
11. Trichopoulou A, Kouris-Blazos A, Wahlqvist ML, et al.Diet and overall survival in elderly people. *BMJ* 1995;**311**:1457–60.
12. Trichopoulou A, Costacou T, Bamia C, et al.Adherence to a Mediterranean diet and survival in a Greek population. *N Engl J Med* 2003;**348**:2599–26
13. Trichopoulou A, Bamia C, Norat T, et al.Modified Mediterranean diet and survival after myocardial infarction: The EPIC-Elderly Study. *Eur J Epidemiol* 2007;**22**:871–81.
14. Martnez-Gonzalez MA, de la Fuente-Arrillaga C, Nunez-Cordoba JM, et al.Adherence to Mediterranean diet and risk of developing diabetes: prospective cohort study. *BMJ* 2008;**336**:1348–51.
15. Nunez-Cordoba JM, Valencia-Serrano F, Toledo E, Alfonso A, Martinez-Gonzalez MA. The Mediterranean Diet and Incidence of Hypertension: The Seguimiento Universidad de Navarra (SUN) Study. *Am J Epidemiol* 2009;**169**:339–46.
16. Psaltopoulou T, Naska A, Orfanos P, Trichopoulos D, Mountokalakis T, Trichopoulou A. Olive oil, the Mediterranean diet, and arterial blood pressure: the Greek European Prospective Investigation into Cancer and Nutrition (EPIC) study. *Am J Clin Nutr* 2004;**80**:1012–8.
17. Rumawas ME, Meigs JB, Dwyer JT, McKeown NM, Jacques PF. Mediterranean-style dietary pattern, reduced risk of metabolic syndrome traits, and incidence in the Framingham Offspring Cohort. *Am J Clin Nutr* 2009;**90**:1608–14.
18. Buckland G, Bach A, Serra-Majem L. Obesity and the Mediterranean diet: a systematic review of observational and interventional studies. *Obes Rev* 2008;**9**:582–93.
19. Kastorini CM, Milionis HJ, Esposito K, Giugliano D, Goudevenos JA, Panagiotakos DB. The effect of Mediterranean diet on metabolic syndrome and its components: a meta-analysis of 50 studies and 534,906 individuals. *J Am Coll Cardiol* 2011;**57**:1299–1313.
20. Estruch R et al. Primary prevention of Cardiovascular Disease with a Mediterranean diet (NEJM)
21. Barzi F, Woodward M, Marfisi RM, et al.Mediterranean diet and all-causes mortality after myocardial infarction: results from the GISSI-Prevenzione trial. *Eur J Clin Nutr* 2003;**57**:604–11.
22. de Lorgeril M, Renaud S, Mamelle N, et al.Mediterranean alpha-linolenic acid-rich diet in secondary prevention of coronary heart disease. *Lancet* 1994;**343**:1454–9.
23. de Lorgeril M, Salen P, Martin JL, et al.Effect of a Mediterranean type of diet on the rate of cardiovascular complications in patients with coronary artery disease. Insights into the cardioprotective effect of certain nutriments. *J Am Coll Cardiol* 1996;**28**:1103–8.
24. de Lorgeril M, Salen P, Martin J, Monjaud I, Delaye J, Mamelle N. Mediterranean diet, traditional risk factors and the rate of cardiovascular complications after myocardial infarction: final report of the Lyon diet heart study. *Circulation* 1999;**99**:799–85.
25. Helsing E, Trichopoulou A. The Mediterranean diet and food culture: a symposium. *Eur J Clin Nutr* 1989;**43**(suppl1):1–92.
26. Nestle E. Mediterranean diets: science and policy implications. *Am J Clin Nutr* 1995;**61**(suppl 6):1313–1427.
27. Serra-Majem L, Helsing E. Changing patterns of fat intake in Mediterranean countries. *Eur J Clin Nutr* 1993;**47**(suppl1):1–100.
28. Willett WC, Sacks F, Trichopoulou A, Drescher G, Ferro-Luzzi A, Helsing E, et al.Mediterranean diet pyramid: a cultural model for healthy eating. *Am J Clin Nutr* 1995;**61** (suppl 6)::1402–6.
29. Higgins JPT, Green S (editors). Cochrane Handbook for Systematic Reviews of Interventions Version 5.1.0 [updated March 2011]. The Cochrane Collaboration 2011. Available from www.cochrane-handbook.org.
30. Woodward M (ed). *Epidemiology: design and data analysis*. 2nd edn. Boca Raton, Florida, USA: Chapman and Hall/CRC Press 2005.
31. Egger M, Davey Smith G, Schneider M, Minder C. Bias in meta-analysis detected by a simple, graphical test. *BMJ* 1997; **315**: 629-34.

**APPENDIX**

**Search strategy**

MEDLINE & CENTRAL

1. exp Clinical Trial/

2. exp Random Allocation/

3. exp Single Blind Method/

4. exp Double Blind Method/

5. (random$ adj5 trial$).tw.

6. (random$ adj5 allocation$).tw.

7. (Blind$ adj5 method$).tw.

8. or/1-7

9. diet, mediterranean/

10. (mediterranean adj3 diet$).tw.

11. (mediterranean adj3 food$).tw.

12. exp wine/

13. red wine$.tw.

14. exp seafood/

15. seafood$.tw.

16. cheese$.tw.

17. exp cheese/

18. exp fruit/

19. or/9-18

20. 8 and 19

EMBASE

#1 'randomized controlled trial'/exp OR 'randomized controlled trial'

#2 'clinical trial (topic)'/exp OR 'clinical trial (topic)'

#3 'mediterranean diet'/exp OR 'mediterranean diet'

#4 'red wine'/exp OR 'red wine'

#5 'red wine'/exp OR 'red wine'

#6 'seafood'/exp OR 'seafood'

#7 'nut'/exp OR 'nut'

#8 'cheese'/exp OR 'cheese'

#9 1 or 2

#10 or/4-8

#11 9 and 10
