# Supplementary material for: Effects of the Mediterranean Diet on Cardiovascular Outcomes—A Systematic Review and Meta-Analysis
Source: PLoS One. 2016 Aug 10;11(8):e0159252. doi: 10.1371/journal.pone.0159252 (PMC4980102; doi:10.1371/journal.pone.0159252)
Supplement: S2 File — (DOCX) [file pone.0159252.s004.docx]

**S2 File. Search strategy**

MEDLINE & CENTRAL

1. exp Clinical Trial/

2. exp Random Allocation/

3. exp Single Blind Method/

4. exp Double Blind Method/

5. (random$ adj5 trial$).tw.

6. (random$ adj5 allocation$).tw.

7. (Blind$ adj5 method$).tw.

8. or/1-7

9. diet, mediterranean/

10. (mediterranean adj3 diet$).tw.

11. (mediterranean adj3 food$).tw.

12. exp wine/

13. red wine$.tw.

14. exp seafood/

15. seafood$.tw.

16. cheese$.tw.

17. exp cheese/

18. exp fruit/

19. or/9-18

20. 8 and 19

EMBASE

#1 'randomized controlled trial'/exp OR 'randomized controlled trial'

#2 'clinical trial (topic)'/exp OR 'clinical trial (topic)'

#3 'mediterranean diet'/exp OR 'mediterranean diet'

#4 'red wine'/exp OR 'red wine'

#5 'red wine'/exp OR 'red wine'

#6 'seafood'/exp OR 'seafood'

#7 'nut'/exp OR 'nut'

#8 'cheese'/exp OR 'cheese'

#9 1 or 2

#10 or/4-8

#11 9 and 10
