# Supplementary material for: Effects of the Mediterranean Diet on Cardiovascular Outcomes—A Systematic Review and Meta-Analysis
Source: PLoS One. 2016 Aug 10;11(8):e0159252. doi: 10.1371/journal.pone.0159252 (PMC4980102; doi:10.1371/journal.pone.0159252)
Supplement: S3 File — (RTF) [file pone.0159252.s005.rtf]

S3 File. List of rejected articles after full text review 

1.	Abu-Amsha Caccetta R, Burke V, Mori TA, Beilin LJ, Puddey IB, Croft KD. Red wine polyphenols, in the absence of alcohol, reduce lipid peroxidative stress in smoking subjects. Free radical biology & medicine. 2001;30(6):636-42.

2.	Barona J, Jones JJ, Kopec RE, Comperatore M, Andersen C, Schwartz SJ, et al. A Mediterranean-style low-glycemic-load diet increases plasma carotenoids and decreases LDL oxidation in women with metabolic syndrome. The Journal of nutritional biochemistry. 2012;23(6):609-15.

3.	Bemelmans WJ, Broer J, de Vries JH, Hulshof KF, May JF, Meyboom-De Jong B. Impact of Mediterranean diet education versus posted leaflet on dietary habits and serum cholesterol in a high risk population for cardiovascular disease. Public health nutrition. 2000;3(3):273-83.

4.	Ben-Avraham S, Harman-Boehm I, Schwarzfuchs D, Shai I. Dietary strategies for patients with type 2 diabetes in the era of multi-approaches; review and results from the Dietary Intervention Randomized Controlled Trial (DIRECT). Diabetes research and clinical practice. 2009;86 Suppl 1:S41-8.

5.	Bravo-Herrera MD, Lopez-Miranda J, Marin C, Gomez P, Gomez MJ, Moreno JA, et al. Tissue factor expression is decreased in monocytes obtained from blood during Mediterranean or high carbohydrate diets. Nutrition, metabolism, and cardiovascular diseases : NMCD. 2004;14(3):128-32.

6.	Bullo M, Amigo-Correig P, Marquez-Sandoval F, Babio N, Martinez-Gonzalez MA, Estruch R, et al. Mediterranean diet and high dietary acid load associated with mixed nuts: effect on bone metabolism in elderly subjects. Journal of the American Geriatrics Society. 2009;57(10):1789-98.

7.	Carruba G, Granata OM, Pala V, Campisi I, Agostara B, Cusimano R, et al. A traditional Mediterranean diet decreases endogenous estrogens in healthy postmenopausal women. Nutrition and cancer. 2006;56(2):253-9.

8.	Castagnetta L, Granata OM, Cusimano R, Ravazzolo B, Liquori M, Polito L, et al. The Mediet Project. Annals of the New York Academy of Sciences. 2002;963:282-9.

9.	Corle DK, Sharbaugh C, Mateski DJ, Coyne T, Paskett ED, Cahill J, et al. Self-rated quality of life measures: effect of change to a low-fat, high-fiber, fruit and vegetable enriched diet. Annals of behavioral medicine : a publication of the Society of Behavioral Medicine. 2001;23(3):198-207.

10.	De Lorgeril M, Salen P, Martin JL, Mamelle N, Monjaud I, Touboul P, et al. Effect of a mediterranean type of diet on the rate of cardiovascular complications in patients with coronary artery disease. Insights into the cardioprotective effect of certain nutriments. Journal of the American College of Cardiology. 1996;28(5):1103-8.

11.	de Lorgeril M, Salen P, Martin JL, Monjaud I, Delaye J, Mamelle N. Mediterranean diet, traditional risk factors, and the rate of cardiovascular complications after myocardial infarction: final report of the Lyon Diet Heart Study. Circulation. 1999;99(6):779-85.

12.	Defoort C, Vincent-Baudry S, Lairon D. Effects of 3-month Mediterranean-type diet on postprandial TAG and apolipoprotein B48 in the Medi-RIVAGE cohort. Public health nutrition. 2011;14(12a):2302-8.

13.	Diaz-Lopez A, Bullo M, Martinez-Gonzalez MA, Guasch-Ferre M, Ros E, Basora J, et al. Effects of Mediterranean diets on kidney function: a report from the PREDIMED trial. American journal of kidney diseases : the official journal of the National Kidney Foundation. 2012;60(3):380-9.

14.	Djuric Z, Ren J, Blythe J, VanLoon G, Sen A. A Mediterranean dietary intervention in healthy American women changes plasma carotenoids and fatty acids in distinct clusters. Nutrition research (New York, NY). 2009;29(3):156-63.
15.	Elhayany A, Lustman A, Abel R, Attal-Singer J, Vinker S. A low carbohydrate Mediterranean diet improves cardiovascular risk factors and diabetes control among overweight patients with type 2 diabetes mellitus: a 1-year prospective randomized intervention study. Diabetes, obesity & metabolism. 2010;12(3):204-9.

16.	Esposito K, Maiorino MI, Ciotola M, Di Palo C, Scognamiglio P, Gicchino M, et al. Effects of a Mediterranean-style diet on the need for antihyperglycemic drug therapy in patients with newly diagnosed type 2 diabetes: a randomized trial. Annals of internal medicine. 2009;151(5):306-14.

17.	Estruch R, Martinez-Gonzalez MA, Corella D, Basora-Gallisa J, Ruiz-Gutierrez V, Covas MI, et al. Effects of dietary fibre intake on risk factors for cardiovascular disease in subjects at high risk. Journal of epidemiology and community health. 2009;63(7):582-8.

18.	Estruch R, Martinez-Gonzalez MA, Corella D, Salas-Salvado J, Ruiz-Gutierrez V, Covas MI, et al. Effects of a Mediterranean-style diet on cardiovascular risk factors: a randomized trial. Annals of internal medicine. 2006;145(1):1-11.

19.	Fernandez-Real JM, Bullo M, Moreno-Navarrete JM, Ricart W, Ros E, Estruch R, et al. A Mediterranean diet enriched with olive oil is associated with higher serum total osteocalcin levels in elderly men at high cardiovascular risk. The Journal of clinical endocrinology and metabolism. 2012;97(10):3792-8.

20.	Fernemark H, Jaredsson C, Bunjaku B, Rosenqvist U, Nystrom FH, Guldbrand H. A randomized cross-over trial of the postprandial effects of three different diets in patients with type 2 diabetes. PloS one. 2013;8(11):e79324.

21.	Fraser A, Abel R, Lawlor DA, Fraser D, Elhayany A. A modified Mediterranean diet is associated with the greatest reduction in alanine aminotransferase levels in obese type 2 diabetes patients: results of a quasi-randomised controlled trial. Diabetologia. 2008;51(9):1616-22.

22.	Heinrich H, Goetze O, Menne D, Iten PX, Fruehauf H, Vavricka SR, et al. Effect on gastric function and symptoms of drinking wine, black tea, or schnapps with a Swiss cheese fondue: randomised controlled crossover trial. BMJ (Clinical research ed). 2010;341:c6731.

23.	Itsiopoulos C, Brazionis L, Kaimakamis M, Cameron M, Best JD, O'Dea K, et al. Can the Mediterranean diet lower HbA1c in type 2 diabetes? Results from a randomized cross-over study. Nutrition, metabolism, and cardiovascular diseases : NMCD. 2011;21(9):740-7.

24.	Jones JL, Fernandez ML, McIntosh MS, Najm W, Calle MC, Kalynych C, et al. A Mediterranean-style low-glycemic-load diet improves variables of metabolic syndrome in women, and addition of a phytochemical-rich medical food enhances benefits on lipoprotein metabolism. Journal of clinical lipidology. 2011;5(3):188-96.

25.	Khymenets O, Fito M, Covas MI, Farre M, Pujadas MA, Munoz D, et al. Mononuclear cell transcriptome response after sustained virgin olive oil consumption in humans: an exploratory nutrigenomics study. Omics : a journal of integrative biology. 2009;13(1):7-19.

26.	Lankinen M, Schwab U, Kolehmainen M, Paananen J, Poutanen K, Mykkanen H, et al. Whole grain products, fish and bilberries alter glucose and lipid metabolism in a randomized, controlled trial: the Sysdimet study. PloS one. 2011;6(8):e22646.

27.	McTiernan A, Wactawski-Wende J, Wu L, Rodabough RJ, Watts NB, Tylavsky F, et al. Low-fat, increased fruit, vegetable, and grain dietary pattern, fractures, and bone mineral density: the Women's Health Initiative Dietary Modification Trial. The American journal of clinical nutrition. 2009;89(6):1864-76.

28.	Mena MP, Sacanella E, Vazquez-Agell M, Morales M, Fito M, Escoda R, et al. Inhibition of circulating immune cell activation: a molecular antiinflammatory effect of the Mediterranean diet. The American journal of clinical nutrition. 2009;89(1):248-56.
29.	Mezzano D, Leighton F, Strobel P, Martinez C, Marshall G, Cuevas A, et al. Mediterranean diet, but not red wine, is associated with beneficial changes in primary haemostasis. European journal of clinical nutrition. 2003;57(3):439-46.

30.	Michalsen A, Lehmann N, Pithan C, Knoblauch NT, Moebus S, Kannenberg F, et al. Mediterranean diet has no effect on markers of inflammation and metabolic risk factors in patients with coronary artery disease. European journal of clinical nutrition. 2006;60(4):478-85.

31.	Murie-Fernandez M, Irimia P, Toledo E, Martinez-Vila E, Buil-Cosiales P, Serrano-Martinez M, et al. Carotid intima-media thickness changes with Mediterranean diet: a randomized trial (PREDIMED-Navarra). Atherosclerosis. 2011;219(1):158-62.

32.	Paniagua JA, de la Sacristana AG, Sanchez E, Romero I, Vidal-Puig A, Berral FJ, et al. A MUFA-rich diet improves posprandial glucose, lipid and GLP-1 responses in insulin-resistant subjects. Journal of the American College of Nutrition. 2007;26(5):434-44.

33.	Papandreou C, Schiza SE, Bouloukaki I, Hatzis CM, Kafatos AG, Siafakas NM, et al. Effect of Mediterranean diet versus prudent diet combined with physical activity on OSAS: a randomised trial. The European respiratory journal. 2012;39(6):1398-404.

34.	Papandreou C, Schiza SE, Tzatzarakis MN, Kavalakis M, Hatzis CM, Tsatsakis AM, et al. Effect of Mediterranean diet on lipid peroxidation marker TBARS in obese patients with OSAHS under CPAP treatment: a randomised trial. Sleep & breathing = Schlaf & Atmung. 2012;16(3):873-9.

35.	Razquin C, Martinez JA, Martinez-Gonzalez MA, Salas-Salvado J, Estruch R, Marti A. A 3-year Mediterranean-style dietary intervention may modulate the association between adiponectin gene variants and body weight change. European journal of nutrition. 2010;49(5):311-9.

36.	Shike M, Latkany L, Riedel E, Fleisher M, Schatzkin A, Lanza E, et al. Lack of effect of a low-fat, high-fruit, -vegetable, and -fiber diet on serum prostate-specific antigen of men without prostate cancer: results from a randomized trial. Journal of clinical oncology : official journal of the American Society of Clinical Oncology. 2002;20(17):3592-8.

37.	Singh RB, Ghosh S, Singh R. Effects on serum lipids of adding fruits and vegetables to prudent diet in the Indian Experiment of Infarct Survival (IEIS). Cardiology. 1992;80(3-4):283-93.

38.	Singh RB, Niaz MA, Ghosh S, Singh R, Rastogi SS. Effect on mortality and reinfarction of adding fruits and vegetables to a prudent diet in the Indian experiment of infarct survival (IEIS). Journal of the American College of Nutrition. 1993;12(3):255-61.

39.	Singhal S, Gupta R, Goyle A. Comparison of antioxidant efficacy of vitamin E, vitamin C, vitamin A and fruits in coronary heart disease: a controlled trial. The Journal of the Association of Physicians of India. 2001;49:327-31.
40.	Sola R, Fito M, Estruch R, Salas-Salvado J, Corella D, de La Torre R, et al. Effect of a traditional Mediterranean diet on apolipoproteins B, A-I, and their ratio: a randomized, controlled trial. Atherosclerosis. 2011;218(1):174-80.

41.	Stachowska E, Gutowska I, Strzelczak A, Wesolowska T, Safranow K, Ciechanowski K, et al. The use of neural networks in evaluation of the direction and dynamics of changes in lipid parameters in kidney transplant patients on the Mediterranean diet. Journal of renal nutrition : the official journal of the Council on Renal Nutrition of the National Kidney Foundation. 2006;16(2):150-9.

42.	Stendell-Hollis NR, Thompson PA, West JL, Wertheim BC, Thomson CA. A comparison of Mediterranean-style and MyPyramid diets on weight loss and inflammatory biomarkers in postpartum breastfeeding women. Journal of women's health (2002). 2013;22(1):48-57.

43.	Velazquez-Lopez L, Santiago-Diaz G, Nava-Hernandez J, Munoz-Torres AV, Medina-Bravo P, Torres-Tamayo M. Mediterranean-style diet reduces metabolic syndrome components in obese children and adolescents with obesity. BMC pediatrics. 2014;14:175.

44.	Walsh PC. Lack of effect of a low-fat, high-fruit, -vegetable, and -fiber diet on serum prostate-specific antigen of men without prostate cancer: results from a randomized trial. The Journal of urology. 2003;169(4):1592-3.

45.	Zazpe I, Sanchez-Tainta A, Estruch R, Lamuela-Raventos RM, Schroder H, Salas-Salvado J, et al. A large randomized individual and group intervention conducted by registered dietitians increased adherence to Mediterranean-type diets: the PREDIMED study. Journal of the American Dietetic Association. 2008;108(7):1134-44; discussion 45.
